# Chemopreventive Properties of Black Raspberries and Strawberries in Esophageal Cancer Review

**DOI:** 10.3390/antiox11091815

**Published:** 2022-09-15

**Authors:** Ni Shi, Tong Chen

**Affiliations:** 1 Division of Medical Oncology, Department of Internal Medicine, The Ohio State University, 1800 Cannon Drive, 13th Floor, Columbus, OH 43210, USA; 2 Comprehensive Cancer Center, The Ohio State University, Columbus, OH 43210, USA

**Keywords:** black raspberry, strawberry, esophageal cancer, cancer prevention, anthocyanins

## Abstract

Esophageal cancer is one of the most fetal malignancies in the world. Esophageal squamous cell carcinoma (SCC) and esophageal adenocarcinoma (AC) are two main types of esophageal cancer and each with distinct epidemiological, etiological and histopathological characteristics. The continued global prevalence of tobacco use and alcohol consumption, coupled with limited intake of fresh fruits and vegetables, ensures that esophageal cancer will remain one of the major health threats. In addition to promoting quitting smoking and alcohol abuse, one of the strategies of cancer prevention is to identify foods, food components, or dietary patterns that can prevent or delay the onset of esophageal cancer. A food-based approach has the advantage of a complex of mixtures of bioactive components simultaneously targeting multiple processes in carcinogenesis. We have employed a preclinical rodent model of esophageal SCC to assess the effects of black raspberries (BRB) and strawberries. Our investigations demonstrate that BRB and strawberries are potent inhibitors of esophageal cancer. To prepare for this review, a literature search was performed to screen BRB and strawberries against esophageal cancer using electronic databases from PubMed, Science Direct and Google Scholar. Search was conducted covering the period from January 2000 to June 2022. Our present review has provided a systematic review about chemopreventive effects of BRB and strawberries in esophageal cancer by collecting and compiling diverse research findings from the above sources. In this review, we discussed the anti-tumor potentials of BRB and strawberries in esophageal SCC and esophageal AC separately. For each cancer type, we discuss animal models and research findings from both animal bioassays and human clinical studies. We also discuss the potential mechanisms of action of berries and their key bioactive components.

## 1. Introduction

Esophageal cancer is a major public health concern worldwide [1]. The two main types of esophageal cancer are esophageal squamous cell carcinoma (SCC; 90%) and esophageal adenocarcinoma (AC; 5%) [2]. The overall survival rates of esophageal cancer have not changed substantially during the past several decades despite improvements regarding detection, staging of this disease and aggressive multimodality intervention. The data from the National Cancer Institute’s Surveillance, Epidemiology, and End Results (SEER) Program showed that in the United States, although esophageal AC was more prevalent in Caucasians, esophageal SCC predominates in African Americans over Caucasians by five folds in male and three folds in female [3].

The risk factors of esophageal SCC are multiple including tobacco use, alcohol consumption, intake of mycotoxins-contaminated food, deficiencies in nutrients, and hot soup or hot tea caused thermal injuries [4]. Even though esophageal AC is not the predominant type of esophageal cancer, the incidence and mortality rates of AC are rising in both Europe and North America [5]. Gastroesophageal reflux disease, tobacco consumption and obesity are significant risk factors for esophageal AC [5].

Carcinogenesis is a complex process with interactions among genetics, diet, physical activity, other lifestyle and environmental factors [6]. It should be noted that nutritional factors contribute to about 30% of cancer mortality worldwide [7]. The twenty-four-hour dietary recall data from National Health and Nutrition Examination Survey suggested that 73% of Americans do not meet the minimal USDA guidelines for fruit consumption (2 servings) and 65% did not meet the guidelines for vegetables (3 servings) [8]. The overall 5-year survival rate of esophageal cancer in the United States is only 13%, which is close to the rates in countries and regions where the incidence of esophageal cancer is high [1]. Thus, chemoprevention of esophageal cancer becomes the cornerstone as a means of cancer control. A number of human trials of esophageal cancer prevention were conducted in international populations at high-risk for the disease. However, only limited results have been obtained from these studies, and to date, they have been negative or only marginally suggestive of positive effects. Natural food products play an invaluable role in cancer prevention because of low toxicity and reliable effectiveness. Chemoprevention through dietary intervention would be a logical and practical approach to manage esophageal precancerous disease.

Certain vitamins, minerals and dietary components have been reported to reduce cancer risk. For example, the clinical benefits of vitamin E for breast cancer prevention and beta-carotene for lung cancer are well-known [9,10]. However, these single vitamins and minerals showed controversial results in clinical trials [10]. Recently, whole food-based prevention strategies are of particular interest because a complex of mixtures of bioactive phytochemicals could potentially target multiple processes in esophageal carcinogenesis with minimal or no toxicities. For example, green tea showed potential prevention effects for oral [11], skin [12], cervical [13], prostate [14], upper gastrointestinal tract [15], hepatocellular [16] and lung cancers [17], as well as breast cancer in premenopausal women [18]. Soy and tomato significantly inhibited prostate cancer [19,20]. Curcumin showed prevention effects against colon cancer [21]. Broccoli sprout beverages reduced the risk for liver cancer [22].

Black raspberries (BRB) and strawberries contain a reservoir of healthy contents, such as flavonoid, vitamins, minerals, phytosterols and phytochemicals. The health effects of berries have been studied by the scientific community [23,24]. Our laboratory has conducted extensive research in the past two decades elucidating the mechanism(s) through which BRB and strawberries impact esophageal, oral and colon carcinogenesis. The bioactivity of BRB has been suggested in numerous pilot intervention trials in humans including Barrett’s esophagus [25], oral dysplasia [26] and colorectal cancer [27]. In preclinical animal studies, the strawberry diet exhibits cancer preventive potential in the oral cavity [28], breast [29], lung [30], and esophagus [31]. In a pilot study, we demonstrate that 6-month strawberry treatment reverses the precancerous histopathological changes in patients with esophageal dysplasia [32].

In this review, we summarize the efficiency of BRB and strawberries for esophageal cancer prevention. We discuss the impact of berries on esophageal SCC and AC separately. We also elucidate molecular events and signaling pathways modulated by BRB and strawberries in esophageal cancer prevention. Finally, we discuss the photochemical profiles of BRB and strawberries and the role of anthocyanins as the major components in berries which may be responsible for cancer prevention effects of berries.

## 2. Chemoprevention of BRB and Strawberries in Esophageal SCC

### 2.1. Animal Models of Esophageal SCC

The initiation and progression of esophageal SCC is the result of interaction among multiple factors including environmental and genetic factors. The esophageal histopathological change from normal to carcinoma is a progressive sequence involving hyperplasia and different grades of dysplasia [33,34]. The progression of dysplasia is closely associated with the increased risk of developing esophageal SCC. The natural history of esophageal SCC offers multiple opportunities for assessment and intervention. One of the strategies of cancer prevention is to block the transformation from dysplasia to cancer [35]. Chemoprevention, therefore, is a feasible approach and it may have special relevance in former tobacco smokers and consumers of alcohol who remain at higher risk for esophageal cancer development than non-users, and in the areas or workplaces where people are exposed to carcinogen in the environment.

It is remarkable that an animal model, which mimics the neoplastic progression occurring in humans, tremendously enhance the development of strategies for the chemoprevention of esophageal cancer. The animal model of nitrosamine-induced esophageal cancer is an invaluable and practicable system to study esophageal carcinogenesis and chemoprevention [36,37,38,39,40,41,42]. *N*-nitrosomethylbenzylamine (NMBA) is by far the most potent inducer of tumors in the rat esophagus through metabolic activation [42]. The key metabolism process of NMBA is the formation of *O*^6^-methylguanine. The *O*^6^-methylguanine adduction leads to single base mispairing in DNA and finally induces carcinogenesis. In a typical animal carcinogenesis protocol, NMBA is injected subcutaneously at various doses (e.g., 0.25, 0.3 or 0.5 mg/kg b.w.) for a certain of period (e.g., 5, 10 or 15 weeks) [38,43]. Esophageal hyperplasia, dysplasia and papilloma are produced in the esophagi of NMBA-treated rats. The sequence of change from esophageal hyperplasia to cancer in animals mimic the histopathological progression in human, which could be captured during esophageal endoscopy [44]. In addition to the NMBA-induced esophageal SCC rat model, there are other animal models for esophageal cancer research, such as the xenograft model [45], mice with zinc-deficiency [46] or mice treated with carcinogen, 4-nitroquinoline 1-oxide (4-NQO) [47].

### 2.2. Efficacy of BRB in NMBA-Induced Esophageal SCC

**Figure 1:**
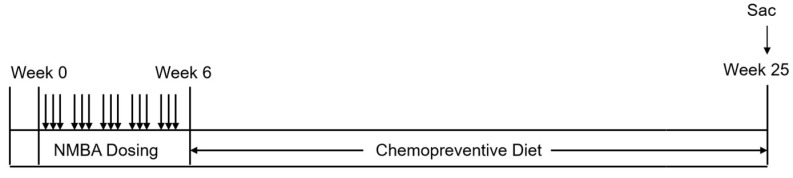
Anti-tumor progression protocol. Rats are treated with NMBA at either 0.25, 0.30 or 0.5 mg/kg b.w. three times a week for five weeks. Rats are given either control diet or experimental diet containing preventive agent(s) for the entire bioassay. Rats are scarified at Week 25.

The efficacy of BRB in prevention of esophageal SCC has been first investigated by our research group since 2000. The berry research received a lot of attention and was extended by the scientific community in recent years. To obtain freeze-dried berry powder, whole berries are picked, shipped frozen, freeze-dried in a commercial grade food lyophilizer, grinded into a powder and stored at −20 °C until shipped frozen to the research labs. At the Ohio State University (OSU), we analyze berry components at OSU Nutrient and Phytochemical Analytics Shared Resource (NPASR). It is crucial that the berries are chemically and biologically uniform. Based on our experience from the past decades, the yearly change in berry components is less than 20% comparing to those that are picked from the same farm source in a different year. In an animal bioassay, the experimental diet (BRB diet) is the control diet (AIN-76A diet) containing BRB and is prepared fresh weekly. In a standard anti-tumor progression study, Fischer-344 rats are randomly assigned to different experimental groups and placed on AIN-76A diet (control diet) at the beginning of the study. Rats are treated with NMBA three times per week for five weeks. The chemopreventive diet (experimental diet) is given after NMBA treatment. At the end of bioassay rats are sacrificed, and esophageal tissues are collected (Figure 1). In our previous study, we found that BRB reduced the tumor incidence and tumor multiplicity in rats treated with NMBA [39].

In our previous investigations, we found that the levels of cyclooxygenase 2 (COX-2) and inducible nitric oxide synthase (iNOS) in esophageal epithelium of rats treated with NMBA were higher than those in control animals [37,48]. We subsequently demonstrated that L-748706, a selective COX-2 inhibitor, and S,S′-1,4-phenylene-bis(1,2-ethanediyl)bis-isothiourea (PBIT), a selective iNOS inhibitor, significantly inhibit NMBA-induced rat esophageal tumorigenesis [43,49]. To compare the effects of BRB and chemically pure agents, we conducted another animal study in which we tested a combination of celecoxib, a selective COX-2 inhibitor, and PBIT in comparison to a diet enriched with lyophilized BRB in NMBA-induced rat esophageal tumorigenesis. The primary outcomes were tumor development and growth of esophageal premalignant lesions. Our data indicated that rats fed experimental diet containing BRB or combination of celecoxib + PBIT exhibited a greater percentage of normal and hyperplasia compared to animals fed the control diet. Rats fed BRB- or celecoxib + PBIT had less low-grade dysplasia, high-grade dysplasia and papilloma compared to animals fed control diet [40]. Our study demonstrates that BRB exhibits a more potent effect in inhibiting esophageal carcinogenesis than the combination of celecoxib and PBIT.

In a study conducted by Pan et al., F344 rats were first injected with NMBA and then fed a control diet or 5% BRB. To obtain both esophageal precancerous tissues and tumors, rats were sacrificed at Weeks 9, 15 and 35, respectively [50]. DNA microarray was performed on RNA samples isolated from rat esophagi. The data showed that among 1181 genes induced by NMBA treatment, BRB significantly modulate 428 genes including pro-inflammatory cytokines, such as chemokine (C-C motif) ligand 2 (CCL2), S100A8 (also known as MRP8), and IL-19 [50]. In addition, BRB modulated genes involved inflammation, cell differentiation, cell cycle, angiogenesis, and carbohydrate and lipid metabolism [51,52,53]. In another study conducted by Huang et al., the aim was to investigate DNA methylation in NMBA-treated rats and the effect of BRB in preventing aberrant DNA methylation if it occurs during esophageal tumorigenesis [54]. Their data showed that inhibition of esophageal SCC by BRB was associated with DNA methyltransferases 1 (DNMT 1), DNMT 3b, and methylation of secreted frizzled-related protein 4 (Sfrp4) [54].

It should be noted that an attempt was made to investigate the potential cancer therapeutic effects of BRB in the NMBA-rat animal model. In this study, NMBA was given once a week for 15 weeks. Instead of starting the berry diet at the initiation stage of carcinogenesis in a typical chemoprevention study, rats were fed BRB (5%, 10% or 20%) at Week 19 when they had already developed multiple tumors in their esophagi. This study showed that BRB increased the survival of NMBA-treated rats in a dose dependent manner, however, it was not significant [55]. NMBA dosing, BRB concentration and treatment windows are key elements to accurately measure tumor outcome and survival in this animal model. Efforts are needed to establish a more practical animal bioassay protocol to study the cancer therapeutic effects of berries.

### 2.3. Efficacy of Strawberries in NMBA-Induced Esophageal SCC

We evaluated the efficacy of strawberries for their anti-initiation effects in NMBA-induced esophageal SCC. At a 10% concentration in the diet, administration of lyophilized strawberries resulted in a more than 50% reduction in tumor numbers [31]. In addition, 5% and 10% lyophilized strawberries significantly reduced DNA adduct formation and the incidence of leukoplakia, a dysplastic premalignant lesion, by 52 and 65%, respectively, compared to rats treated with NMBA and fed control diet [31].

In another study conducted by Pan et al., the effect of strawberries alone and in combination with aspirin against esophageal cancer was investigated in rats [56]. They found that strawberry alone significantly decreased tumor multiplicity and the combination treatment exhibited a more potent effect [56]. To identify more berry types for esophageal cancer prevention, Stoner et al. evaluated the effects of seven different berries including BRB, strawberries, blueberries, red raspberries, noni, açaí and wolfberries, in animal model of esophageal SCC. Their data showed that all berry types were about equally potent in suppressing esophageal tumorigenesis. More experiments are needed to further investigate the mechanisms of action of these berries [57].

### 2.4. Mechanisms of Action of Berries against Esophageal SCC

As a natural product with multiple components, BRB has the potential to inhibit esophageal SCC development via multiple mechanisms. Using high-throughput gene expression and pathway cluster analysis platforms, an abundance of genes modulated by BRB to inhibit esophageal SCC carcinogenesis were identified. In a microarray analysis performed by Wang et al. [51], their clustering analysis found that BRB modulated genes involved in carbohydrate/lipid metabolism, arachidonic acid metabolism, cell proliferation and differentiation, apoptosis, cell adhesion, angiogenesis, tissue invasion and metastasis, and inflammation.

The inducible nitric oxide synthase (iNOS) is a calcium and calmodulin independent enzyme, which catalyzes the conversion of L-arginine to citrulline with the high production of nitric oxide (NO) [58]. Up-regulation of iNOS has been reported in several types of cancer including breast [59], head and neck [60], lung [61], colon [62], melanoma [63], prostate [64], as well as esophageal cancer [65]. COX-2 is induced by certain stimuli including cytokines, growth factors, tumor promoters and hormones [66]. COX-2 catalyzes the formation of prostanoids including prostaglandin E_2_ (PGE_2_), which affects cell growth, differentiation, cell cycles, angiogenesis and metastasis [67]. Overexpression of COX-2 has been reported in cancers of colon [68], gastric [69], breast [70], skin [71], pancreas [72], lung [73], head and neck [74], urinary bladder [75], and esophagus [76]. Our data suggest that BRB reduces iNOS and COX-2 expression, and decreases production of PGE_2_ and total nitrite in rats treated with NMBA plus BRB when compared to those treated with NMBA only [38].

Angiogenesis could promote tumor growth and expand opportunities for tumor cells to transfer to the general circulation and thus metastasize. Vascular endothelial growth factor (VEGF) is the major angiogenesis factor during carcinogenesis and tumor metastasis. VEGF is upregulated in various cancers including lung [77], breast [78], head and neck [79], thyroid [80], gastric [81], uterus [82], prostate [83], colon [84], and esophagus [85]. Microvessel density (MVD) is an index of new micro vessel growth and is commonly used to assess angiogenesis in tissues. The positive correlation between VEGF and MVD is observed in human esophageal SCC [86]. In our study, we found that BRB significantly suppressed VEGF and MVD in rats fed experimental diet when compared to animals fed control diet [39].

Oxidant stress is a state in which by-products of oxygen consumption, reactive metabolites, reactive oxygen species (ROS) and antioxidants are imbalanced, which causes macromolecules damage and then tumor formation [87,88]. In rat esophageal SCC, the increased production of iNOS causes oxidative stress [37,58]. In clinical research, 8-hydroxy-2′-deoxyguanosine (8-OHdG) is used to measure the oxidative damage to DNA. Its level in serum of patients with esophageal SCC is higher compared to that in healthy volunteers [89]. Two critical enzymatic systems in producing and decomposing ROS are glutathione peroxidase (GPx) and superoxide dismutase 2 (SOD_2_) [90]. Our study demonstrates that BRB increases GPx and SOD_2_ and reduces the production of 8-oxoguanine (8-OxoG), hydrogen peroxide (H_2_O_2_) and lipid hydroperoxide (LPO) [91]. Our data indicate that BRB reverses oxidative stress in NMBA-induced esophageal tumorigenesis in rats.

We further investigated the efficacy of BRB compared to celecoxib + PBIT. Our data show that BRB exhibits more potent effects not only on the expression and activity of COX-2 and iNOS in treated esophagi, but also on their upstream regulated pathways including mitogen-activated protein kinases (MAPK), Nuclear factor kappa B (NFκB) and AKT [40]. The efficacy of BRB in esophageal cancer prevention may be due to the co-activities of BRB’s parent phytochemicals, their metabolic intermediates and ultimate metabolites. In this first parallel investigation of BRB and a combination of two synthesized drugs, we observed that BRB was more potent than the combination in preventing esophageal cancer.

Moreover, in a study performed by Peiffer et al. [92], BRB is shown to decrease expression of the proinflammatory cytokine Interleukin-1β (IL-1β), pentraxin-3 (PTX3), soluble epoxide hydrolase (sEH) and increase expression of the anti-inflammatory cytokine, IL-10 and natural killer cell activator, IL-12. BRB decreases infiltration of macrophages and neutrophils into esophageal tissues, thus enhancing the immune systems and eliminating the cancer cells [92].

Our data demonstrate that having a rich profile of diverse phytochemicals, BRB suppress carcinogenesis through multiple mechanisms of action which may be synergetic and interactive.

### 2.5. Chemoprevention of Esophageal SCC in Human

Esophageal carcinogenesis is a multistage process and a transformation from normal squamous epithelium to basal cell hyperplasia, dysplasia and ultimate carcinoma may take several decades. Dysplasia is a precancerous lesion of esophageal SCC with three histopathological grades: mild, moderate and severe. It can be accurately visualized and targeted during esophageal endoscopy and biopsy. Patients with esophageal dysplasia have an increased risk for developing esophageal SCC. The observational epidemiology study reported that the incidence of esophageal SCC in patients with mild dysplasia or moderate dysplasia is about 25% and 50%, respectively, in a period of decades [35]. In clinic, the lesions of esophageal severe dysplasia are usually removed by endoscopic mucosal resection or argon plasma coagulation. Mild and moderate dysplasia are usually monitored by health professionals based on the current standard of care. Since dysplasia can be visualized and measured during routine endoscopy, we could use it as a surrogate endpoint or primary outcome in a chemoprevention/therapeutic clinical trial.

We conducted a single-arm pilot clinical study to assess if dietary strawberries could impact precancerous growth in patients with esophageal dysplastic lesions [32]. The study subjects who met the inclusion criteria were consented and assigned to one of the two treatment arms, 30 g/day or 60 g/day, for six months. Esophageal dysplastic lesions were collected during endoscopy before and after strawberry treatment. Histopathological analysis indicated that dietary strawberries (60 g/day) reduced the histopathological progression of esophageal precancerous lesions in patients with esophageal dysplasia. The low dose strawberry treatment (30 g/day) did not significantly suppress the progression of precancerous growth. In addition, the mechanistic studies show that strawberries significantly inhibited cell proliferation, proinflammatory cytokines and NFκB signaling in human esophageal mucosa [32].

## 3. Chemoprevention of BRB on Esophageal AC

### 3.1. Animal Model of Esophageal AC

Gastroesophageal reflux and its progression to premalignant lesions, Barrett’s esophagus are significant risk factors for esophageal AC [93,94]. Gastrectomy with esophagojejunal anastomosis or esophagoduodenal anastomosis in rodents can induce major biliary reflux which mimics Barrett’s esophagus and subsequent progression to esophageal AC in humans [5,95,96,97]. The esophagoduodenal anastomosis (EDA) is a procedure which is carried out in rats under general anesthesia. It involves an anastomosis of the distal esophagus with duodenum and a removal of entire stomach. The resections are conducted at 2 mm above the gastroesophageal junction and 3 mm distal to the pylorus. The EDA procedure is shown to induce reflux in male rats and produce esophageal AC in 40% of rats within 6 months [97]. The progress of carcinogenesis is longer and tumor incidence is lower comparing to NMBA-induced esophageal SCC animal model, in which about 50% and 100% of NMBA-treated rats develop tumors at Weeks 15 and 25, respectively [38,39,43].

### 3.2. Efficacy and Mechanisms of Action of BRB against Esophageal AC

In the EDA model, oxidative damage is identified as the major risk factor for developing esophageal AC. In a short-term study, the BRB diet shows no effect on the development of esophagitis in the EDA model [95]. In another study to assess the effect of 2.5% BRB in inhibiting esophageal tumorigenesis in the EDA model, the incidences of reflux esophagitis, intestinal metaplasia and esophageal AC are not different among animals fed control or BRB diet [97]. BRB significantly increases the malondialdehyde concentration. BRB also increases the level of antioxidant enzyme MnSOD and decreases total SOD activity, but without significance. These studies suggest that 2.5% BRB is not effective in the prevention of reflux-induced esophageal AC in the EDA rat model. The preclinical investigations of chemopreventive studies with berries are summarized in Table 1.

### 3.3. Chemoprevention of Esophageal AC in Human

Barrett’s esophagus is a precancerous state in which the normal squamous epithelium of the distal esophagus changes to intestinal-like columnar epithelium, and is a result of chronic exposure to gastroesophageal reflux. Patients with Barrett’s esophagus have a 30–40-fold higher risk of developing esophageal AC compared to general populations [98]. In the United States, the prevalence of Barrett’s esophagus is increasing and about 7% Barrett’s esophagus patients with high-grade dysplasia develop to esophageal AC every year [99]. Therefore, a surveillance strategy for early detection of dysplasia in patients with Barrett’s esophagus is needed.

**Figure 2:**
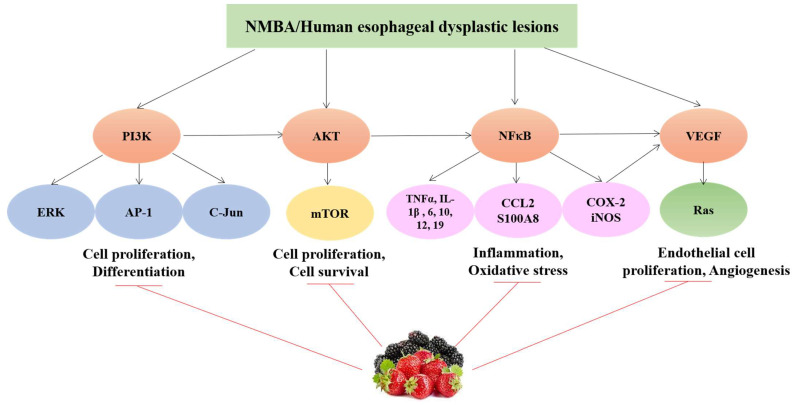
Possible mechanisms of action of BRB and strawberries in NMBA-induced esophageal cancer in animals and in pilot clinical trials.

In a pilot study, the effect of BRB was evaluated in patients with Barrett’s esophagus [25,100]. Twenty patients were recruited in the study and given BRB 32 g (for female) or 45 g (for male) per day for 6 months. Urine samples were collected from each study subject for a 3-h period in the morning at Weeks 0, 12 and 26. A total of 10 patients completed the 6-month BRB treatment. The Barrett’s tongue was 2.9 cm long (2.0–8.0 cm) at the baseline and it was not changed by 6-month BRB treatment. However, the levels of lipid peroxidation markers, 8-epi-prostaglandin F2α (8-Iso-PGF2) and 8-hydroxy-2-deoxyguanosine (8-OHdG), were significantly reduced by BRB in urine samples collected from the patients who completed the 26-week treatment. The clinical investigations of chemopreventive studies with berries are highlighted in Table 2. The mechanisms of action of BRB and strawberries in esophageal cancer prevention are summarized in Figure 2.

## 4. Bioactive Components in BRB and Strawberries

### 4.1. Phytochemical Profiles of BRB and Strawberry

To profile the phytochemical contents in freeze-dried BRB and strawberries that were administered in our previous preclinical studies (NMBA-rat esophageal SCC) and pilot human clinical trial, high-performance liquid chromatography/accurate mass/tandem mass spectrometry (HPLC-MS/MS) was performed at three wavelengths (260, 355 and 500 nm) to identify and quantify BRB and strawberry phytochemicals [101]. We identified and quantified 20 phytochemicals. These compounds are classified into four categories: anthocyanins, ellagitannin, ellagic acid (EA) and derivatives, and flavonols. We identified that anthocyanins account for 58.4% by dry weight of the phenolics in strawberry powder [102]. Pelargonidin-3-glucoside (P3G) is the main anthocyanin (41.1%) in strawberries. We also note that the second main anthocyanin in strawberries is pelargonidin malonyl glucoside (9.38% by dry weight) and its health effect has not been fully investigated. We also identified agrimoniin, the main ellagitannin, as the second most abundant phenolic by dry weight (16.2%) in strawberry powder. In addition, kaempferol counts for 2.2% by dry weight. Unlike in strawberry powder, anthocyanins contribute 84.2% by dry weight of the phenolics in BRB powder [40]. The ellagitannins are 11.5%, EA and derivatives are 0.7%, and flavonols are 3.6%, which counts about 15.8% of non-anthocyanin phenolics. In accordance with previously reported data, the main anthocyanin in BRB is cyaniding-3-rutinoside (C3R; (58.2%). The other anthocyanins are cyaniding-3-xylorutinoside (C3X), cyaniding-3-glucoside (C3G), cyaniding-3-sambubioside (C3S) and pelargonidin rutinoside, which count 18.2%, 4.9%, 1% and 0.8% by dry weight, respectively.

### 4.2. Anthocyanins in Esophageal Cancer Prevention

Anthocyanins are naturally occurring pigmentation polyphenolic compounds in fruits and vegetables, which are responsible for their rich and vibrant color. Both BRB and strawberries are rich in anthocyanins and contain multiple types of anthocyanin compounds. Numerous studies have been performed to evaluate berry’s parent anthocyanins and their metabolites in NMBA-induced esophageal SCC in rats. In a study conducted by Peiffer et al., the esophageal precancerous lesions and tumors were induced by NMBA injection in rats [92]. The experimental diets were a control diet containing BRB, anthocyanins-faction of BRB, or an anthocyanin metabolite, protocatechuic acid (PCA). The plasma and esophageal tissues were collected at Weeks 15, 25 and 35, respectively, for analyzing inflammatory biomarkers. Their results demonstrated that all three experimental diets modulated proinflammatory cytokines including IL-1β, IL-10, and IL-12. Moreover, all three experimental diets suppressed macrophages and neutrophils to infiltrate into the esophagus [92]. The experimental diet containing BRB was more effective than the experimental diet containing anthocyanins or PCA [103]. The similar results were observed in another animal bioassay in which an anthocyanin-rich fraction of berries was assessed [104].

In a study conducted by Wang et al., rats were treated with NMBA and fed experimental diet containing residue fraction of BRB, strawberries or blueberries [105]. The esophageal tissues were collected by the end of bioassay at Week 30. The overall objective of this study was to assess if a berry component, ellagitannins, contributes to cancer preventive effects of berries. Their data showed that all three experimental diets were almost equally potent in reducing tumor development, even though the amount of ellagitannins was different in these three types of berries. Therefore, they concluded that ellagitannins may not be the key bioactive component in berries [105].

## 5. Conclusions

Both BRB and strawberries are effective against esophageal SCC but not esophageal AC in animal models. In a pilot single-arm study we reported for the first time that dietary strawberries significantly inhibit progression of esophageal dysplasia in humans. The synergistic anticancer activities of BRB and strawberries against esophageal SCC are associated with reducing cell proliferation, angiogenesis, inflammation and tumor innate immunity, enhancing cell differentiation and adhesion, and promoting apoptosis. Anthocyanins, the major phenolic components in BRB and strawberries by dry weight, and PCA, the metabolite of anthocyanins, exhibit the similar prevention effects against esophageal SCC as BRB. However, the anthocyanins-rich fractions are a mixer of multiple different anthocyanin compounds. Further studies are needed to identify and investigate the single bioactive berry component and its structure-activity in esophageal cancer prevention. In addition, the pharmacokinetic studies are also needed to elucidate absorption, distribution, metabolism, and excretion of bioactive components in BRB and strawberries. Future investigations also need to assess and compare the efficacy of whole-berry to: (1) single or combination of berry phytochemicals or metabolites; (2) combination of berry compounds with phytochemicals from other foods; and (3) combination of whole-berry and pharmaceutical drugs. The research on esophageal cancer with berries needs to be continuously explored. It will pave the way for the development of natural-food based strategies against esophageal cancer, which may include whole-food approach and development of formulated drugs.

## Figures and Tables

**Table 1:** Chemoprevention of esophageal cancer with berries in animal models.

Berry Type	Animal Model	Endpoint	Findings	References
Black raspberries	NMBA-induced tumorigenesis	Esophageal SCC	Decreased tumor incidence and multiplicity; reduced pro-inflammatory mediators and ni-trosative stress; and suppressed PI3K/AKT and NFkB signaling	[38,39,40,42,51]
Strawberries	NMBA-induced tumorigenesis	Esophageal SCC	Decreased tumor incidence and multiplicity; and reduced DNA adduct formation	[31,56,57]
Black raspberries	Esophagoduodenal anastomosis (EDA) model	Esophageal reflux and AC	Increased the malondialdehyde concentration and MnSOD; and decreased total SOD activity, but without significance	[95,96,97]

**Table 2:** Chemopreventive study with berries in humans.

Berry Type	Study Subject	Treatment	Endpoint	Findings	Reference
Strawberries	Patients with esophageal dysplasia	30 g/day or 60 g/day for 6 months	Histopathological change	Inhibited the progression of pre-cancerous growth by reducing pro-inflammatory mediators, NFkB signaling and cell proliferation	[32]
Black raspberries	Patients with Barrett’s esophagus	32 g/day (female) or 45 g/day (male) for 6 months	Histopatho-logical change	Did not change the histopathology of Barrett’s but reduced oxidative stress	[25,100]

## References

[1] Sung H., Ferlay J., Siegel R.L., Laversanne M., Soerjomataram I., Jemal A., Bray F. (2021). Global Cancer Statistics 2020: GLOBOCAN Estimates of Incidence and Mortality Worldwide for 36 Cancers in 185 Countries. CA Cancer J. Clin..

[2] Trivers K.F., Sabatino S.A., Stewart S.L. (2008). Trends in esophageal cancer incidence by histology, United States, 1998–2003. Int. J. Cancer.

[3] Duggan M.A., Anderson W.F., Altekruse S., Penberthy L., Sherman M.E. (2016). The Surveillance, Epidemiology, and End Results (SEER) Program and Pathology: Toward Strengthening the Critical Relationship. Am. J. Surg. Pathol..

[4] Prabhu A., Obi K.O., Rubenstein J.H. (2014). The synergistic effects of alcohol and tobacco consumption on the risk of esophageal squamous cell carcinoma: A meta-analysis. Am. J. Gastroenterol..

[5] Rustgi A.K., El-Serag H.B. (2014). Esophageal carcinoma. N. Engl. J. Med..

[6] Anand P., Kunnumakkara A.B., Sundaram C., Harikumar K.B., Tharakan S.T., Lai O.S., Sung B., Aggarwal B.B. (2008). Cancer is a preventable disease that requires major lifestyle changes. Pharm. Res..

[7] Key T.J., Bradbury K.E., Perez-Cornago A., Sinha R., Tsilidis K.K., Tsugane S. (2020). Diet, nutrition, and cancer risk: What do we know and what is the way forward?. BMJ.

[8] Bail J., Meneses K., Demark-Wahnefried W. (2016). Nutritional Status and Diet in Cancer Prevention. Semin. Oncol. Nurs..

[9] Lee I.M., Cook N.R., Gaziano J.M., Gordon D., Ridker P.M., Manson J.E., Hennekens C.H., Buring J.E. (2005). Vitamin E in the primary prevention of cardiovascular disease and cancer: The Women’s Health Study: A randomized controlled trial. JAMA.

[10] Harvie M. (2014). Nutritional supplements and cancer: Potential benefits and proven harms. Am. Soc. Clin. Oncol. Educ. Book.

[11] Ramshankar V., Krishnamurthy A. (2014). Chemoprevention of oral cancer: Green tea experience. J. Nat. Sci. Biol. Med..

[12] Katiyar S.K. (2011). Green tea prevents non-melanoma skin cancer by enhancing DNA repair. Arch. Biochem. Biophys..

[13] Zou C., Liu H., Feugang J.M., Hao Z., Chow H.H., Garcia F. (2010). Green tea compound in chemoprevention of cervical cancer. Int. J. Gynecol. Cancer.

[14] Miyata Y., Shida Y., Hakariya T., Sakai H. (2019). Anti-Cancer Effects of Green Tea Polyphenols Against Prostate Cancer. Molecules.

[15] Ren J.S., Freedman N.D., Kamangar F., Dawsey S.M., Hollenbeck A.R., Schatzkin A., Abnet C.C. (2010). Tea, coffee, carbonated soft drinks and upper gastrointestinal tract cancer risk in a large United States prospective cohort study. Eur. J. Cancer.

[16] Ni C.X., Gong H., Liu Y., Qi Y., Jiang C.L., Zhang J.P. (2017). Green Tea Consumption and the Risk of Liver Cancer: A Meta-Analysis. Nutr. Cancer.

[17] Fritz H., Seely D., Kennedy D.A., Fernandes R., Cooley K., Fergusson D. (2013). Green tea and lung cancer: A systematic review. Integr. Cancer Ther..

[18] Shrubsole M.J., Lu W., Chen Z., Shu X.O., Zheng Y., Dai Q., Cai Q., Gu K., Ruan Z.X., Gao Y.T. (2009). Drinking green tea modestly reduces breast cancer risk. J. Nutr..

[19] Applegate C.C., Rowles J.L., Ranard K.M., Jeon S., Erdman J.W. (2018). Soy Consumption and the Risk of Prostate Cancer: An Updated Systematic Review and Meta-Analysis. Nutrients.

[20] Chen J., Song Y., Zhang L. (2013). Lycopene/tomato consumption and the risk of prostate cancer: A systematic review and meta-analysis of prospective studies. J. Nutr. Sci. Vitaminol..

[21] Pricci M., Girardi B., Giorgio F., Losurdo G., Ierardi E., Di Leo A. (2020). Curcumin and Colorectal Cancer: From Basic to Clinical Evidences. Int. J. Mol. Sci..

[22] Kensler T.W., Chen J.G., Egner P.A., Fahey J.W., Jacobson L.P., Stephenson K.K., Ye L., Coady J.L., Wang J.B., Wu Y. (2005). Effects of glucosinolate-rich broccoli sprouts on urinary levels of aflatoxin-DNA adducts and phenanthrene tetraols in a randomized clinical trial in He Zuo township, Qidong, People’s Republic of China. Cancer Epidemiol. Biomark. Prev..

[23] Kula M., Krauze-Baranowska M. (2016). Rubus occidentalis: The black raspberry—Its potential in the prevention of cancer. Nutr. Cancer.

[24] Battino M., Forbes-Hernandez T.Y., Gasparrini M., Afrin S., Cianciosi D., Zhang J., Manna P.P., Reboredo-Rodriguez P., Varela Lopez A., Quiles J.L. (2019). Relevance of functional foods in the Mediterranean diet: The role of olive oil, berries and honey in the prevention of cancer and cardiovascular diseases. Crit. Rev. Food Sci. Nutr..

[25] Kresty L.A., Frankel W.L., Hammond C.D., Baird M.E., Mele J.M., Stoner G.D., Fromkes J.J. (2006). Transitioning from preclinical to clinical chemopreventive assessments of lyophilized black raspberries: Interim results show berries modulate markers of oxidative stress in Barrett’s esophagus patients. Nutr. Cancer.

[26] Mallery S.R., Stoner G.D., Larsen P.E., Fields H.W., Rodrigo K.A., Schwartz S.J., Tian Q., Dai J., Mumper R.J. (2007). Formulation and in-vitro and in-vivo evaluation of a mucoadhesive gel containing freeze dried black raspberries: Implications for oral cancer chemoprevention. Pharm. Res..

[27] Wang L.S., Arnold M., Huang Y.W., Sardo C., Seguin C., Martin E., Huang T.H., Riedl K., Schwartz S., Frankel W. (2011). Modulation of genetic and epigenetic biomarkers of colorectal cancer in humans by black raspberries: A phase I pilot study. Clin. Cancer Res..

[28] Zhu X., Xiong L., Zhang X., Shi N., Zhang Y., Ke J., Sun Z., Chen T. (2015). Lyophilized strawberries prevent 7,12-dimethylbenz[α]anthracene (DMBA)-induced oral squamous cell carcinogenesis in hamsters. J. Funct. Foods.

[29] Somasagara R.R., Hegde M., Chiruvella K.K., Musini A., Choudhary B., Raghavan S.C. (2012). Extracts of strawberry fruits induce intrinsic pathway of apoptosis in breast cancer cells and inhibits tumor progression in mice. PLoS ONE.

[30] Balansky R., Ganchev G., Iltcheva M., Kratchanova M., Denev P., Kratchanov C., Polasa K., D’Agostini F., Steele V.E., De Flora S. (2012). Inhibition of lung tumor development by berry extracts in mice exposed to cigarette smoke. Int. J. Cancer.

[31] Carlton P.S., Kresty L.A., Siglin J.C., Morse M.A., Lu J., Morgan C., Stoner G.D. (2001). Inhibition of N-nitrosomethylbenzylamine-induced tumorigenesis in the rat esophagus by dietary freeze-dried strawberries. Carcinogenesis.

[32] Chen T., Yan F., Qian J., Guo M., Zhang H., Tang X., Chen F., Stoner G.D., Wang X. (2012). Randomized phase II trial of lyophilized strawberries in patients with dysplastic precancerous lesions of the esophagus. Cancer Prev. Res..

[33] Anani P.A., Gardiol D., Savary M., Monnier P. (1991). An extensive morphological and comparative study of clinically early and obvious squamous cell carcinoma of the esophagus. Pathol. Res. Pract..

[34] Kuwano H., Watanabe M., Sadanaga N., Ikebe M., Mori M., Sugimachi K. (1993). Squamous epithelial dysplasia associated with squamous cell carcinoma of the esophagus. Cancer Lett..

[35] Wang G.Q., Abnet C.C., Shen Q., Lewin K.J., Sun X.D., Roth M.J., Qiao Y.L., Mark S.D., Dong Z.W., Taylor P.R. (2005). Histological precursors of oesophageal squamous cell carcinoma: Results from a 13 year prospective follow up study in a high risk population. Gut.

[36] Beer D.G., Stoner G.D. (1998). Clinical models of chemoprevention for the esophagus. Hematol. Oncol. Clin. N. Am..

[37] Chen T., Stoner G.D. (2004). Inducible nitric oxide synthase expression in N-nitrosomethylbenzylamine (NMBA)-induced rat esophageal tumorigenesis. Mol. Carcinog..

[38] Chen T., Hwang H., Rose M.E., Nines R.G., Stoner G.D. (2006). Chemopreventive properties of black raspberries in N-nitrosomethylbenzylamine-induced rat esophageal tumorigenesis: Down-regulation of cyclooxygenase-2, inducible nitric oxide synthase, and c-Jun. Cancer Res..

[39] Chen T., Rose M.E., Hwang H., Nines R.G., Stoner G.D. (2006). Black raspberries inhibit N-nitrosomethylbenzylamine (NMBA)-induced angiogenesis in rat esophagus parallel to the suppression of COX-2 and iNOS. Carcinogenesis.

[40] Shi N., Riedl K.M., Schwartz S.J., Zhang X., Clinton S.K., Chen T. (2016). Efficacy comparison of lyophilised black raspberries and combination of celecoxib and PBIT in prevention of carcinogen-induced oesophageal cancer in rats. J. Funct. Foods.

[41] Shi N., Yu H., Chen T. (2019). Inhibition of esophageal cancer growth through the suppression of PI3K/AKT/mTOR signaling pathway. Oncol. Targets Ther..

[42] Kresty L.A., Morse M.A., Morgan C., Carlton P.S., Lu J., Gupta A., Blackwood M., Stoner G.D. (2001). Chemoprevention of esophageal tumorigenesis by dietary administration of lyophilized black raspberries. Cancer Res..

[43] Chen T., Nines R.G., Peschke S.M., Kresty L.A., Stoner G.D. (2004). Chemopreventive effects of a selective nitric oxide synthase inhibitor on carcinogen-induced rat esophageal tumorigenesis. Cancer Res..

[44] Shi N., Jin F., Zhang X., Clinton S.K., Pan Z., Chen T. (2014). Overexpression of human β-defensin 2 promotes growth and invasion during esophageal carcinogenesis. Oncotarget.

[45] Lee N.P., Chan C.M., Tung L.N., Wang H.K., Law S. (2018). Tumor xenograft animal models for esophageal squamous cell carcinoma. J. Biomed. Sci..

[46] Fong L.Y., Jiang Y., Farber J.L. (2006). Zinc deficiency potentiates induction and progression of lingual and esophageal tumors in p53-deficient mice. Carcinogenesis.

[47] Tang X.H., Knudsen B., Bemis D., Tickoo S., Gudas L.J. (2004). Oral cavity and esophageal carcinogenesis modeled in carcinogen-treated mice. Clin. Cancer Res..

[48] Carlton P.S., Gopalakrishnan R., Gupta A., Liston B.W., Habib S., Morse M.A., Stoner G.D. (2002). Piroxicam is an ineffective inhibitor of N-nitrosomethylbenzylamine-induced tumorigenesis in the rat esophagus. Cancer Res..

[49] Stoner G.D., Qin H., Chen T., Carlton P.S., Rose M.E., Aziz R.M., Dixit R. (2005). The effects of L-748706, a selective cyclooxygenase-2 inhibitor, on N-nitrosomethylbenzylamine-induced rat esophageal tumorigenesis. Carcinogenesis.

[50] Pan P., Dombkowski A.A., Wang L.S., Stoner G.D. (2018). A nutrigenetic approach for investigating the chemopreventive effects of black raspberries during the development of preneoplastic esophagi in rats. J. Berry Res..

[51] Wang L.S., Dombkowski A.A., Seguin C., Rocha C., Cukovic D., Mukundan A., Henry C., Stoner G.D. (2011). Mechanistic basis for the chemopreventive effects of black raspberries at a late stage of rat esophageal carcinogenesis. Mol. Carcinog..

[52] Stoner G.D., Dombkowski A.A., Reen R.K., Cukovic D., Salagrama S., Wang L.S., Lechner J.F. (2008). Carcinogen-altered genes in rat esophagus positively modulated to normal levels of expression by both black raspberries and phenylethyl isothiocyanate. Cancer Res..

[53] Lechner J.F., Reen R.K., Dombkowski A.A., Cukovic D., Salagrama S., Wang L.S., Stoner G.D. (2008). Effects of a black raspberry diet on gene expression in the rat esophagus. Nutr. Cancer.

[54] Huang Y.W., Gu F., Dombkowski A., Wang L.S., Stoner G.D. (2016). Black raspberries demethylate Sfrp4, a WNT pathway antagonist, in rat esophageal squamous cell papilloma. Mol. Carcinog..

[55] Stoner G.D., Aziz R.M. (2007). Prevention and therapy of squamous cell carcinoma of the rodent esophagus using freeze-dried black raspberries. Acta Pharmacol. Sin..

[56] Pan P., Peiffer D.S., Huang Y.W., Oshima K., Stoner G.D., Wang L.S. (2018). Inhibition of the development of N-nitrosomethylbenzylamine-induced esophageal tumors in rats by strawberries and aspirin, alone and in combination. J. Berry Res..

[57] Stoner G.D., Wang L.S., Seguin C., Rocha C., Stoner K., Chiu S., Kinghorn A.D. (2010). Multiple berry types prevent N-nitrosomethylbenzylamine-induced esophageal cancer in rats. Pharm. Res..

[58] Aktan F. (2004). iNOS-mediated nitric oxide production and its regulation. Life Sci..

[59] Garrido P., Shalaby A., Walsh E.M., Keane N., Webber M., Keane M.M., Sullivan F.J., Kerin M.J., Callagy G., Ryan A.E. (2017). Impact of inducible nitric oxide synthase (iNOS) expression on triple negative breast cancer outcome and activation of EGFR and ERK signaling pathways. Oncotarget.

[60] Brennan P.A., Dennis S., Poller D., Quintero M., Puxeddu R., Thomas G.J. (2008). Inducible nitric oxide synthase: Correlation with extracapsular spread and enhancement of tumor cell invasion in head and neck squamous cell carcinoma. Head Neck.

[61] Giatromanolaki A., Tsolou A., Daridou E., Kouroupi M., Chlichlia K., Koukourakis M.I. (2020). iNOS Expression by Tumor-Infiltrating Lymphocytes, PD-L1 and Prognosis in Non-Small-Cell Lung Cancer. Cancers.

[62] Gochman E., Mahajna J., Shenzer P., Dahan A., Blatt A., Elyakim R., Reznick A.Z. (2012). The expression of iNOS and nitrotyrosine in colitis and colon cancer in humans. Acta Histochem..

[63] Ding Z., Ogata D., Roszik J., Qin Y., Kim S.H., Tetzlaff M.T., Lazar A.J., Davies M.A., Ekmekcioglu S., Grimm E.A. (2021). iNOS Associates With Poor Survival in Melanoma: A Role for Nitric Oxide in the PI3K-AKT Pathway Stimulation and PTEN S-Nitrosylation. Front. Oncol..

[64] Erlandsson A., Carlsson J., Andersson S.O., Vyas C., Wikstrom P., Andren O., Davidsson S., Rider J.R. (2018). High inducible nitric oxide synthase in prostate tumor epithelium is associated with lethal prostate cancer. Scand J. Urol..

[65] Barani R., Motalleb G., Maghsoudi H. (2016). Evaluation of iNOS Expression in Esophageal Cancer Patients. Gastrointest. Tumors.

[66] Dubois R.N., Abramson S.B., Crofford L., Gupta R.A., Simon L.S., Van De Putte L.B., Lipsky P.E. (1998). Cyclooxygenase in biology and disease. FASEB J..

[67] Smith W.L., DeWitt D.L., Garavito R.M. (2000). Cyclooxygenases: Structural, cellular, and molecular biology. Annu. Rev. Biochem..

[68] Brown J.R., DuBois R.N. (2005). COX-2: A molecular target for colorectal cancer prevention. J. Clin. Oncol..

[69] Wang Z., Chen J.Q., Liu J.L. (2014). COX-2 Inhibitors and Gastric Cancer. Gastroenterol. Res. Pract..

[70] Regulski M., Regulska K., Prukała W., Piotrowska H., Stanisz B., Murias M. (2016). COX-2 inhibitors: A novel strategy in the management of breast cancer. Drug Discov. Today.

[71] Tudor D.V., Bâldea I., Lupu M., Kacso T., Kutasi E., Hopârtean A., Stretea R., Gabriela Filip A. (2020). COX-2 as a potential biomarker and therapeutic target in melanoma. Cancer Biol. Med..

[72] Nath S., Roy L.D., Grover P., Rao S., Mukherjee P. (2015). Mucin 1 Regulates Cox-2 Gene in Pancreatic Cancer. Pancreas.

[73] Sandler A.B., Dubinett S.M. (2004). COX-2 inhibition and lung cancer. Semin. Oncol..

[74] Frejborg E., Salo T., Salem A. (2020). Role of Cyclooxygenase-2 in Head and Neck Tumorigenesis. Int. J. Mol. Sci..

[75] Al-Maghrabi B., Gomaa W., Abdelwahed M., Al-Maghrabi J. (2019). Increased COX-2 Immunostaining in Urothelial Carcinoma of the Urinary Bladder Is Associated with Invasiveness and Poor Prognosis. Anal. Cell Pathol..

[76] Chen J., Wu F., Pei H.L., Gu W.D., Ning Z.H., Shao Y.J., Huang J. (2015). Analysis of the correlation between P53 and Cox-2 expression and prognosis in esophageal cancer. Oncol. Lett..

[77] Frezzetti D., Gallo M., Maiello M.R., D’Alessio A., Esposito C., Chicchinelli N., Normanno N., De Luca A. (2017). VEGF as a potential target in lung cancer. Expert. Opin Ther. Targets.

[78] Sledge G.W. (2005). VEGF-targeting therapy for breast cancer. J. Mammary Gland Biol. Neoplasia.

[79] Vassilakopoulou M., Psyrri A., Argiris A. (2015). Targeting angiogenesis in head and neck cancer. Oral Oncol..

[80] Ceric S., Ceric T., Pojskic N., Bilalovic N., Musanovic J., Kucukalic-Selimovic E. (2020). Immunohistochemical expression and prognostic significance of VEGF-C in well-differentiated thyroid cancer. Acta Endocrinol..

[81] Chen S., Zhang X., Peng J., Zhai E., He Y., Wu H., Chen C., Ma J., Wang Z., Cai S. (2016). VEGF promotes gastric cancer development by upregulating CRMP4. Oncotarget.

[82] Fu K., Zhang L., Liu R., Shi Q., Li X., Wang M. (2020). MiR-125 inhibited cervical cancer progression by regulating VEGF and PI3K/AKT signaling pathway. World J. Surg. Oncol..

[83] Melegh Z., Oltean S. (2019). Targeting Angiogenesis in Prostate Cancer. Int. J. Mol. Sci..

[84] Ahluwalia A., Jones M.K., Matysiak-Budnik T., Tarnawski A.S. (2014). VEGF and colon cancer growth beyond angiogenesis: Does VEGF directly mediate colon cancer growth via a non-angiogenic mechanism?. Curr. Pharm. Des..

[85] Gu H., Qiu W., Shi Y., Chen S., Yin J. (2014). Variant alleles of VEGF and risk of esophageal cancer and lymph node metastasis. Biomarkers.

[86] Shih C.H., Ozawa S., Ando N., Ueda M., Kitajima M. (2000). Vascular endothelial growth factor expression predicts outcome and lymph node metastasis in squamous cell carcinoma of the esophagus. Clin. Cancer Res..

[87] Pérez S., Taléns-Visconti R., Rius-Pérez S., Finamor I., Sastre J. (2017). Redox signaling in the gastrointestinal tract. Free Radic. Biol. Med..

[88] Reuter S., Gupta S.C., Chaturvedi M.M., Aggarwal B.B. (2010). Oxidative stress, inflammation, and cancer: How are they linked?. Free Radic. Biol. Med..

[89] Diakowska D., Lewandowski A., Kopeć W., Diakowski W., Chrzanowska T. (2007). Oxidative DNA damage and total antioxidant status in serum of patients with esophageal squamous cell carcinoma. Hepatogastroenterology.

[90] Nordberg J., Arnér E.S. (2001). Reactive oxygen species, antioxidants, and the mammalian thioredoxin system. Free Radic. Biol. Med..

[91] Shi N., Chen F., Zhang X., Clinton S.K., Tang X., Sun Z., Chen T. (2017). Suppression of Oxidative Stress and NFκB/MAPK Signaling by Lyophilized Black Raspberries for Esophageal Cancer Prevention in Rats. Nutrients.

[92] Peiffer D.S., Wang L.S., Zimmerman N.P., Ransom B.W., Carmella S.G., Kuo C.T., Chen J.H., Oshima K., Huang Y.W., Hecht S.S. (2016). Dietary Consumption of Black Raspberries or Their Anthocyanin Constituents Alters Innate Immune Cell Trafficking in Esophageal Cancer. Cancer Immunol. Res..

[93] Shaheen N., Ransohoff D.F. (2002). Gastroesophageal reflux, barrett esophagus, and esophageal cancer: Scientific review. JAMA.

[94] Nicholson A., Jankowski J. (2011). Acid reflux and oesophageal cancer. Recent Results Cancer Res..

[95] Aiyer H.S., Li Y., Liu Q.H., Reuter N., Martin R.C. (2011). Dietary freeze-dried black raspberry’s effect on cellular antioxidant status during reflux-induced esophagitis in rats. Nutrition.

[96] Garman K.S., Orlando R.C., Chen X. (2012). Review: Experimental models for Barrett’s esophagus and esophageal adenocarcinoma. Am. J. Physiol. Gastrointest. Liver Physiol..

[97] Aiyer H.S., Li Y., Losso J.N., Gao C., Schiffman S.C., Slone S.P., Martin R.C. (2011). Effect of freeze-dried berries on the development of reflux-induced esophageal adenocarcinoma. Nutr. Cancer.

[98] Sharma P. (2009). Clinical practice. Barrett’s esophagus. N. Engl. J. Med..

[99] Rastogi A., Puli S., El-Serag H.B., Bansal A., Wani S., Sharma P. (2008). Incidence of esophageal adenocarcinoma in patients with Barrett’s esophagus and high-grade dysplasia: A meta-analysis. Gastrointest. Endosc..

[100] Kresty L.A., Fromkes J.J., Frankel W.L., Hammond C.D., Seeram N.P., Baird M., Stoner G.D. (2018). A phase I pilot study evaluating the beneficial effects of black raspberries in patients with Barrett’s esophagus. Oncotarget.

[101] Gu J., Ahn-Jarvis J.H., Riedl K.M., Schwartz S.J., Clinton S.K., Vodovotz Y. (2014). Characterization of black raspberry functional food products for cancer prevention human clinical trials. J. Agric. Food Chem..

[102] Shi N., Clinton S.K., Liu Z., Wang Y., Riedl K.M., Schwartz S.J., Zhang X., Pan Z., Chen T. (2015). Strawberry phytochemicals inhibit azoxymethane/dextran sodium sulfate-induced colorectal carcinogenesis in Crj: CD-1 mice. Nutrients.

[103] Peiffer D.S., Zimmerman N.P., Wang L.S., Ransom B.W., Carmella S.G., Kuo C.T., Siddiqui J., Chen J.H., Oshima K., Huang Y.W. (2014). Chemoprevention of esophageal cancer with black raspberries, their component anthocyanins, and a major anthocyanin metabolite, protocatechuic acid. Cancer Prev. Res..

[104] Wang L.S., Hecht S.S., Carmella S.G., Yu N., Larue B., Henry C., McIntyre C., Rocha C., Lechner J.F., Stoner G.D. (2009). Anthocyanins in black raspberries prevent esophageal tumors in rats. Cancer Prev. Res..

[105] Wang L.S., Hecht S., Carmella S., Seguin C., Rocha C., Yu N., Stoner K., Chiu S., Stoner G. (2010). Berry ellagitannins may not be sufficient for prevention of tumors in the rodent esophagus. J. Agric. Food Chem..

